# Notes and News

**Published:** 1892-08-13

**Authors:** 


### Notes and News.

COLLECTED AND COMMUNICATED.

We cannot help regretting that the old Queen's College, Birmingham, is really dead. The medical teaching of the city is for the future to be conducted at the Mason College, dating from September 1st next.

Lately, during the last fortnight in fact, we have been hearing of the riots which have arisen in Russia owing to the cholera epidemic. Mobs have attacked the hospitals, believing blindly the cry of the political agitators and ringleaders that men were being buried in their coffins alive, a horrible notion, likely enough to incense the superstitious population. But a solution of this fallacy is traced in the *Neue Freie Presse* by Dr. Frey, who from personal experience states it as a fact that the bodies of persons who have died from cholera are often seized with muscular contortions three hours after death, and as the contortions have been known to last several hours it is quite possible that this is the origin of the attack on the hospitals and their staff.

The centenary of Percy Bysshe Shelley has come and gone. He was born at Horsham, in Sussex, and that county has taken the lead in organizing a centenary celebration. The opportunity of honouring and perpetuating Shelley's memory in the place of his birth is one of which it was hoped students and lovers of his poetry in every continent would be eager to take advantage. For this purpose an appeal for funds was issued in the confident belief that there must be many who would wish to enable the committee of organisation to give complete and substantial expression to an aim so thoroughly in accord with Shelley's message to the world. There is no concealing the fact, that the celebration, which was not attended by any one of note in the literary world, was a gigantic failure. It is proposed to establish a Shelley library and museum at Horsham. Subscriptions may be sent to the hon. secretaries, J. Stanley Little, Buck's Green, Rudgwick, Horsham, Sussex; and J. J. Robinson, *West Sussex Gazette*, Arundel, Sussex. Cheques should be crossed "London and County Banking Company, Limited, Horsham Branch," and made payable to "The Shelley Memorial Fund."

Last week the Prince of Wales, who is Grand Prior of the Order of St. John of Jerusalem, presented the medals awarded by the Chapter of that Order to those who had distinguished themselves by acts of bravery in saving life. One recipient was a little girl of seven, who received a silver medal for saving a lad from a burning limekiln in Dublin. It is very creditable to the police force that so many of their number were amongst those who received awards.

As was pointed out by the Lieutenant-Governor of the Isle of Man, who presided at the annual meeting of the subscribers to Noble's Isle of Man General Hospital, sorrow and suffering are closely associated with pleasure, paradoxical though the assertion may appear. A fair portion of the increased subscriptions and donations during the past year were the result of the effort made on behalf of the hospital by the Douglas Harriers and the Douglas Cycling Club. The hospital supplies a growing need, and it is satisfactory to know that it receives the support it requires. The Lieutenant-Governor put in a timely reminder of the visitation of cholera in 1833, and of the ravages it then made in the island, and more particularly in Douglas. Of late years the inhabitants have had a fortunate freedom from disease, but there are many dark places in the island, and it is well known that certain parts of Douglas are not the models of cleanliness which sanitarians would desire to see them. It is to be hoped no extra strain will be placed on the hospital during the autumn months, as it has sufficient demands to supply in the ordinary course of events. The institution seems to have been peculiarly fortunate in the number and amount of the legacies which have recently been bequeathed to it.

"We have got free education, and now all I care about is the endowment of old age," was a remark we overheard the other day, and the speaker finished up with: "These sort of social questions which affect everybody seem a great deal more important than whether Ireland gets Home Rule." Most of us feel this endowment of old age to be a weighty problem but one none the less demanding attention, and it is encouraging to find the British Association considered it worthy of a place in their multitudinous discussions. The general opinion, however, in the debate which followed the Reverend Moore Ede's paper on the subject, was against any State interference. The new schemes are all "in the air," but our only old scheme is before us, and somehow it does not commend itself; destitution is the only condition of assistance. The man who has saved a few pounds, will not, if it be known, get any help until his savings are gone, but the gentleman who has taken no care for the morrow in any sense, but who has arrived at old age and incapacity for work, is helped by the parish, and why? Because he is destitute; but the reason of his destitution is hidden. Mr. Lock thinks all will come right when the independence of the individual and the family is strengthened; he also thinks Mr. Ede's scheme, like Mr. Chamberlain's, is "in the air," so we venture to think is the "independence" of most families. Nobody objects to the State endowing destitution as it does in our present outdoor relief; why should it not endow thrift for a change?

Manchester is still in a state of divided opinion as to what they are to do with their Royal Infirmary, and feeling runs high. Owens College is being credited with all sorts of schemes, and what will ultimately be done nobody seems to know; the trustees' meeting certainly was not productive of much, excepting a newspaper warfare, and that is still in progress. It is stated that the reports as to the insanitary state of the infirmary are quite untrue, but statements without statistics are vague and untrustworthy, and we should much like to hear who is right on this point. Certainly with our present knowledge it seems surprising that the only alternative suggested by some should be the erection of a six-storyed building on the old infirmary site. However, the poll which was demanded at Tuesday's meeting will send us a little farther on the road, and as the opposition to the enlargement is pretty general in Manchester, it is probable that the Board of Management will not find a majority of supporters.

With Mr. Edward Cock, who died last week at Kingston-on-Thames, at the ripe age of eighty-seven, the list of the great surgeons who flourished in the earlier part of the century comes to an end. He was the last of Sir Astley Cooper's pupils. Educated at Guy's, he was soon appointed to the staff of that hospital. He was a skilful dissector, and fifty years ago "Cock's Head and Neck" was a popular text book. Sir Astley Cooper said of him that, "if you were to go to Cock in the middle of the night and ask him the origin and insertion of any muscle, he'd tell you without waking up." He was very popular with his dressers, and the students generally; full of stories and professional anecdotes, which he told well, his slight hesitation in speaking adding piquancy to the telling; many of them will long be remembered. He was well known at Kingston, and his death will be regretted in all parts of the world where Guy's men have settled, and none of the staff of that hospital will be held in kinder remembrance than he who was always known as Teddy Cock.

---

That the Ancoats Hospital, situated as it is in the midst of a teeming population of working people, should have an adverse balance is a regrettable, though not an unparalleled, circumstance. It is true the deficit, amounting to about £700, is not a very serious one, but turn which way we will nearly all our most useful institutions seem to be in the same predicament. Much as this hospital has done to alleviate suffering and restore health to the inhabitants of the crowded neighbourhood in which it is situated, that much is as nought compared with what is required to be done. Though the number of cots has been increased during the past year by fourteen, there have on many occasions been as many as four or five applicants for every vacant bed. This is eloquent testimony to the fact that the population has outgrown the hospital, and, we regret to think, the liberality of the public. With the present deficit there seems little likelihood of the Governors being able to provide additional accommodation for patients who are literally waiting at their doors. Manchester has plenty of wealthy people; here is an opportunity for some of them to do good by filling an empty ward with beds.

